# “The integration of aerobic exercise in rehabilitation of a subacute inpatient with spinal cord injury: a case report”

**DOI:** 10.1038/s41394-026-00736-2

**Published:** 2026-05-22

**Authors:** Lorenzo D’Orio, Adriana Semprini, Elisa Barbiani, Francesco Giuseppe Materazzi

**Affiliations:** 1 Bachelor in Physiotherapy, Department of Biomedical and Neuromotor Sciences (DIBINEM), Alma Mater Studiorum-Università di Bologna, 40138 Bologna, Italy; 2 Professional development and implementation of research in healt professions unit, IRCCS Azienda Ospedaliero-Universitaria di Bologna, 40138 Bologna, Italy; 3 Department of Biomedical and Neuromotor Sciences (DIBINEM), Alma Mater Studiorum-Università di Bologna, 40138 Bologna, Italy; 4 Villa Bellombra Hospital, Bologna, Italy; 5 Montecatone Rehabilitation Institute, 40026 Imola, Italy

**Keywords:** Rehabilitation, Quality of life, Outcomes research

## Abstract

**Introduction:**

Spinal cord injury (SCI) is a complex condition that significantly impacts the patient’squality of life. The integration of Aerobic Training (AT) with Conventional Therapy (CT) during rehabilitation is often underestimated, despite its potential to prevent long-term complications. This study aims to evaluate the integration of AT into the CT program of a hospitalized patient with ASIA B tetraplegia.

**Case presentation:**

A 25 year oldindividual with complete motor tetraplegia was admitted to the hospital in the sub-acute phase. AT sessionsintegrated with CT were performed twice daily, 5 days a week including three hydrokinesis and standing frame therapy sessions on weekends. AT was delivered using an upper limb cycle ergometer, consisting of 10 min of work at 20% of predicted heart rate (HR), 5 min of rest, and 5 min of work at 35% of predicted HR. The MRC scale, SCIM scaleand WHO-QOL questionnaire were administered before and after six days of combined CT and AT. Heart rate reserve (HRR), Respiratory rate (RR), rate of perceived exertion (RPE), blood pressure and SpO₂ were measured before and after each AT session.

**Discussion:**

Despite the brief duration of the rehabilitation program, preliminary adaptations were observed in parameters such as RPE. These findings suggest that AT may play a valuable role in rehabilitation protocols for patients with SCI in the subacute phase. However, due to thesingle patient design and short duration of intervention (six-days), further research with larger sample sizes and longer follow-up is necessary to validate these results.

## Introduction

Spinal cord injury (SCI) is a complex clinical condition that has a significant impact on quality of life and lifestyle. In the acute phase following injury, rehabilitation focuses primarily on preventing secondary complications such as pressure ulcers, deep vein thrombosis, ankylosis, muscle contractures, muscle atrophy and subsequently on enhancing mobility and autonomy. Although the integration of Aerobic Training (AT) with Conventional Therapy (CT) has demonstrated considerable potential in improving rehabilitation outcomes; its application during subacute phase remains underutilized. Literature highlights the psychophysical benefits of exercise, including aerobic activity, in healthy individuals, emphasizing that such benefits are most pronounced when exercises are specific, individualized and performed regularly and consistently over time. Recognizing this potential, the first exercise guideline for people with SCI were published in 2011, recommend at least 20 min of moderate-intensity strength and aerobic activity twice a week [1]. This recommendation indicates that adults with SCI may accumulate fitness benefits from activity volumes lower than those advocated for the general population. Nonetheless, some authors argue for higher exercise targets, proposing to perform at least 150 min of aerobic exercise weekly for people with SCI [2]. To obtain additional health benefits, more extensive moderate-intensity physical training exceeding 300 min per week is recommended [1].

Piercy et al. outline general exercise guidelines for adults with chronic health conditions and disabilities that include 150–300 min per week of moderate-intensity, or 75–150 min per week of vigorous-intensity aerobic physical activity.While Ginis et al. provide evidence-based guidelines for exercise in adults with SCI aimed to improve physical fitness, cardiometabolic health, mental health indices and exhibiting a neuroprotective effects in people with SCI [3–6].

In 2018, guidelines clarified the necessary dosage of exercise required to achieve significant improvements in cardiorespiratory capacity and cardiometabolic health among adults with SCI [4, 7]. These guidelines stress that aerobic exercise must meet specific intensity thresholds and training volumes to elicit meaningful physiological adaptations; otherwise, beneficial outcomes are unlikely [4, 8].

Many individuals with SCI tend to reach an inactive and deconditioned physical condition. This can be the result of muscle paralysis, impairments in the autonomic nervous system and wheelchair dependence [8]. Deconditioning can be described as an impaired physical performance, notably affects manual wheelchair propulsion capacity and wheelchair-related physical fitness [8]; leading to reduced cardiovascular health, diminished wheelchair skills, lower physical activity levels, reduced societal participation and compromised quality of life. Contrary to the findings of Hutchinson and Goosey-Tolfrey, which suggest that low-intensity and low-volume aerobic exercise does not result in meaningful physiological adaptations, Scheer and colleagues demonstrated that even low-intensity aerobic exercise performed regularly in a wheelchair (25–40% of heart rate reserve (HRR), 3–5 sessions per week, 14–45 min per session) can enhance physical fitness levels [4]. Notably, beginner level participants following such training protocols demonstrated marked improvements, including a 34% increase in wheelchair-specific peak aerobic work capacity, a 17% increase in sub-maximal fitness and a 31% increase in anaerobic work capacity within seven weeks [8–11].

This case report evaluates the integration of AT into subacute rehabilitation protocol for a hospitalized patient with ASIA B tetraplegia. By applying evidence-based guidelines provided by the American College of Sports Medicine (ACSM) [12], this study proposes a practical and accessible aerobic protocol suitable for spinal units. The study explores the preliminary effects of this approach on functional recovery, fatigue, quality of life, and the prevention of complications. The selected outcome measures were chosen for their clinical relevance and feasibility, although more sensitive metrics such as peak oxygen (VO₂ max) or metabolic equivalents (METs) could improve future studies.

## Case presentation

The study was conducted on a 25 year old individual clinically diagnosed with complete motor tetraplegia at neurological level C6 (AIS-B), according to ASIA/ISCoS International Standards for Neurological Classification of Spinal Cord Injury (ISNCSCI) [13], resulting from a diving accident. Following the injury, the patient completed six-months of intensive rehabilitation in a specialized spinal unit; during which some fluctuations in patient’s mood were observed. Currently, the patient is in the sub-acute phase, characterized by a clinical stability and the absence of autonomic dysreflexia and hypotensive episodes.

The motor assessment shows preserved muscle strength of the deltoids at the upper limb level, bilaterally functional elbow flexors and active, functional forearm pronation-supination. Wrist extensors strength is fully preserved on the right side, while slight hyposthenia (MRC 4/5) is noted on the left side. The tenodesis grasp was achieved during the acute phase of rehabilitation. No active movement is observed in the lower limbs.

Sensory examination reveals impaired pain sensitivity starting from C7, while tactile sensitivity, though impaired, remains present up to T12. Vague sensitivity is present at S3-S4-S5. Osteo-tendinous reflexes are bilaterally vivid, Hoffmann’s reflex is positive bilaterally and the Babinski reflex is spontaneous on the right and evoked on the left.

The CARE (Consensus-based clinical case reporting) guidelines were followed for this report [14]. Treatment was administered at Villa Bellombra, an accredited private hospital in Bologna (https://villabellombra.it/) over six days. The adult patient signed the written informed consent for study participation, including agreement to share clinical and personal data, and photographs for publication purposes.

The daily treatment comprised of two rehabilitation sessions per day, including hydrokinesi therapy, CT combined with AT. Within the rehabilitation treatment in the aquatic pool, passive mobilization techniques and mobilization targeting all four limbs were employed in combination with upper-limb muscle activation strategies and trunk muscle strengthening exercises.

**Fig. 1:**
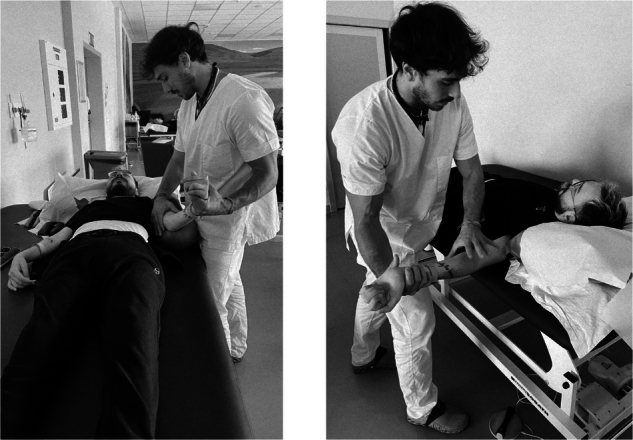
Muscular strengthening exercises were performed on the muscles of the upper limbs and periscapular muscles against resistance and against gravity using concentric, eccentric and isometric contractions.

During CT sessions, strengthening exercises for the upper limbs and periscapular muscles were performed against gravity using resistance equipment such as weights, overballs, swiss balls and elastic bands. These exercises involved concentric, eccentric and isometric contractions (Fig. 1). In addition, training included trunk control exercises in sitting and long-sitting positions, as well as postural transitions techniques. CT was combined with AT, using an upper limb cycle-ergometer; the patient wore an abdominal brace and elasto-compressive stockings throughout the session.

Prior to training the patient was educated on proper motor task execution and encouraged to report any perceived sensations before, during and after the session. This feedback allowed the specialist to modify the training load based on physiological responses, in accordance with the American College of Sport Medicine (ACSM) guidelines for SCI. Monitoring physiological parameters included heart rate (HR), respiratory rate(RR),oxygen saturation (SpO₂), blood pressure(BP) and rate of perceived exertion(RPE). These were measured using a pulse oximeter, sphygmomanometer, stethoscope, smart-watch/manual HR monitoring and Borg scale (6–20). Each parameter was measured at the beginning and end of the 20 min training session.

**Fig. 2:**
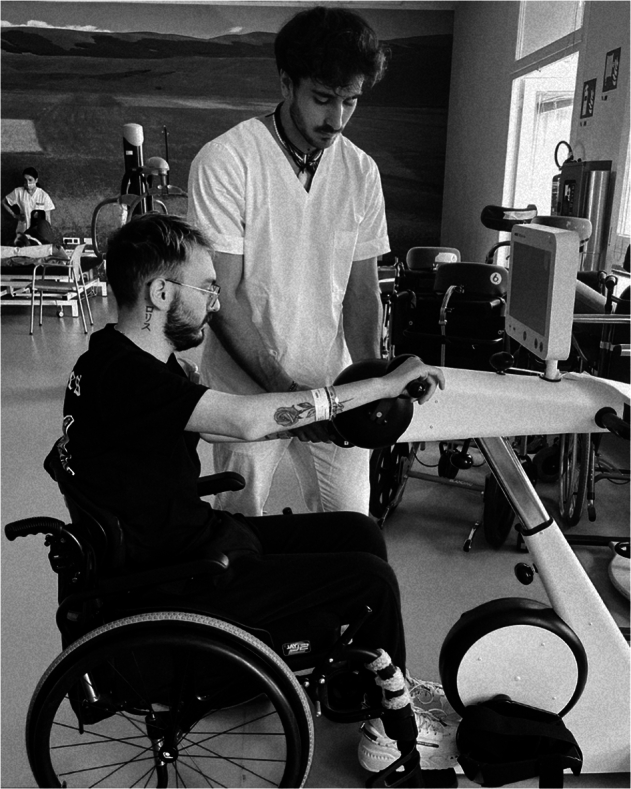
The wheelchair was positioned so that the patient could move his upper limbs as efficiently as possible in front of the cycle-ergometer. The cycle-ergometer had a display that provided data on the quantity of pedal strokes or cyclic movements performed by the limbs, the power each limb provided during the exercise, the total time spent, the distance traveled, the resistance, and the speed at which the exercise was performed.

The cycle ergometer was equipped with a display that provided information on the number of pedal strokes, power produced by each limb, duration, distance, resistance and speed (Fig. 2). The patient remained seated in his wheelchair, positioned in front of the machine to enable efficient movement of the upper limbs, while avoiding full elbow extension during each push on the handle piece, thereby allowing continuous and effective muscle power delivery.

**Fig. 3:**
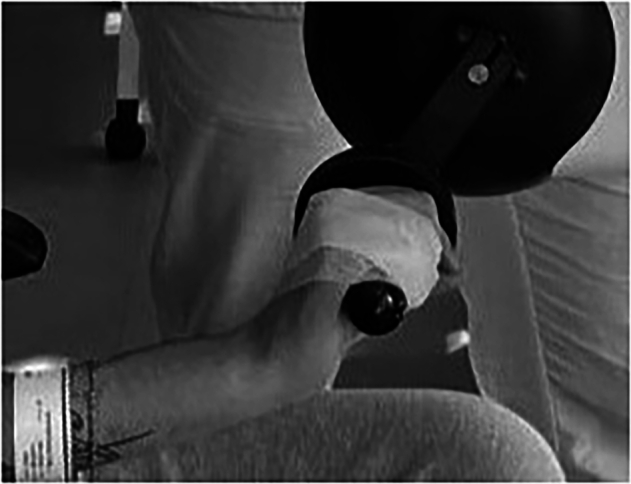
Due to the lack of functional motor control in the patient’s finger and wrist flexor muscles, the hands were taped shut to ensure safety and continuità during the planned training.

The patient’s hands were secured with tape, due to insufficient motor function in the finger and wrist flexors. This setting was essential to ensure safe and continuous engagement in proposed training (Fig. 3).

On the first two days of AT, training began with a resistance equal to 3 watts and speed equal to 20 revolutions per minute (rpm) with initial session duration of 8–10 min and a final duration of 5 min depending on patient’s tolerance. Each session included 5 min of active rest at 0 watts resistance and 3 rpm. This active rest mode was maintained until the end of the week, though rest duration was adjusted as needed.

The dosage of exercise, including resistance, speed and duration, was modified progressively over the 6 days of treatment. By the end, a standard protocol was implemented consisting of 6 min of training (6 watts, 15 rpm), followed by 3–4 min of active rest (0 watts, 3 rpm) and an additional 6 min of training (6 watts, 15 rpm).

Progression of training load was guided by the patient’s perceived exertion and achievement of each session’s specific targeted goals. While ACSM guidelines recommend AT intensity at 40–59% of HRR for this population, observed training sessions achieved HRR values ranging from 31–37%.

To evaluate outcomes, the protocol included the administration of the Medical Research Council (MRC) scale to assess upper limb strength, the Spinal cord independence measure (SCIM) to evaluate functional independence, and the WHO-Quality of Life (WHO-QOL) questionnaire to assess quality of life before and after the combined CT and AT intervention.

## Discussion

This case study aimed to explore the feasibility and potential early benefits of integrating AT during subacute rehabilitation in a patient with AIS B tetraplegia, using an ACSM-based protocol and low-cost, commonly available equipment.

In this case report, HR data collected at the end of each session, demonstrate the following progression from day one to the day six: 98 bpm (~31% HRR), 99 bpm (~36% HRR), 101 bpm (~30%HRR), 103 bpm (~32%HRR), 98 bpm (~31% HRR) and 102 bpm (~37% HRR). These findings suggest that even with a consistent training intensity range, the heart’s ability to supply oxygen to the working muscles during the required activity may have improved, as evidenced by a reduction in the patient’s perceived fatigue. As previously stated, the fluctuation in HR values reflect an acute phase of adaptation, but it is expected that continued aerobic exercise could promote greater cardio-respiratory adaptation, including reduced HR at rest and improved HR response during activity.

RR remained stable during AT sessions but showed a progressive post-exercise decline, particularly by the final session, despite higher training intensities. This trend, despite stable RPE scores, suggests an improvement in exercise tolerance. As reported in Table 1, the average RR during the first three sessions was approximately 21–22 breaths per minute (31–35% HRR), decreasing to 18 breaths per minute by the final day despite higher training intensity (37% HRR). This trend may correlate with perceived exertion on the Borg scale (6–20), which remained at 14–15/20 through out the intervention, classified as “heavy” exertion (~80% HR max; 75–84% VO₂ max) even as the training intensity increased.

**Table 1:** Daily Physiological Parameters and RPE Trend.

	Day 1	Day 2	Day 3	Day 4	Day 5	Day 6
HRR (initial)	54 bpm	45 bpm	51 bpm	53 bpm	55 bpm	48 bpm
HRR (fina	98 bpm	99 bpm	101 bpm (30% HRR)	103 bpm (32% HRR)	98 bpm (31% HRR)	102 bpm
BP (initial)	/	116/70 mmHg	105/60 mmHg	115/70 mmHg	104/68 mmHg	120/80 mmHg
BP (final)	/	98/50 mmHg	98/60 mmHg	90/65 mmHg	95/60 mmHg	115/80 mmHg
SPO_2_ (initial)	98%	98%	98%	98%	98%	98%
SPO_2_ (final)	98%	98%	98%	98%	98%	98%
RPE (initial)	9/20	8/20	8/20	8/20	8/20	6/20
RPE (final)	16/20	14/20	15/20	17/20	15/20	15/20
RR (initial)	/	/	13 rpm	12 rpm	12 rpm	11 rpm
RR (final)	/	/	19 rpm	23 rpm	21 rpm	18 rpm

*HRR* heart rate reserve, *BP* blood pressure, *SPO2* oxygen saturation, *RPE* rating of perceived exertion, *RR* respiratory rate.

Oxygen saturation values remained unchanged throughout the week, both at resting and post-exercise, indicating stable oxygenation during the training. BP was closely monitored as people with SCI are prone to hypotensive episodes. In fact, evidence indicates that both AT and counter-resistance exercise, when performed individually, can elicit acute and chronic reductions in blood pressure [15].

Preliminary adaptations were observed in exertional response parameters, particularly in RR and RPE, highlighting potential early physiological adjustments. Notably by the end of the week, the intensity of AT reached 37% of HRR, yet RPE and RR values were lower compared to the earlier sessions with lesser intensity but the parameters related to physical exertion were higher.While physiological parameters showed signs of early adaptation, functional scores remained unchanged over the brief intervention period. No significant changes were observed in MRC or SCIM scores, which is consistent with the short duration of intervention; however, a slight improvement in WHO-QOL questionnaire suggests a positive trend in psychosocial engagement (Table 2).

**Table 2:** Clinical and Functional Outcome Measures at T0 and T1.

	T0	T1
Medical Research Council (MRC) Scale	biceps brachii 5/5 triceps brachii 2/5 wrist extensors 5/5 wrist flexors 0/5 deltoid 5/5 great pectoral 3-/5	biceps brachii 5/5 triceps brachii 2/5 wrist extensors 5/5 wrist flexors 0/5 deltoid 5/5 great pectoral 3-/5
WHO-QOL	79	81
SCIM	40	40

The MRC (Medical Research Council) scale, and the SCIM (Spinal cord independence measure) scale did not change before and after the short cycle of sessions, as might be expected, however it would be interesting to add them in studies with a larger sample to evaluate the effectiveness of the integration of AT (aerobic training) and CT (conventional training). The WHO-QOL questionnaire improbe slightly but it would be interesting to see the results in the long term and with a larger sample of subjects with acute SCI.

The time course of RR is highly associated with RPE (Borg scale 6–18), especially under conditions of muscle fatigue, increased body temperature, and prior resistance training [16]. As a sensitive parameter to perceived exercise fatigue, RR is considered a key parameter for monitoring physiological responses during and after training [16, 17].

Although it cannot be definitively concluded that the observed changes are due to the six-day intervention, initial data support the potential of AT to positively impact psychophysical well-being and motivation in patients with SCI [18]. While few studies have examined these factors in the SCI population, some authors suggest that exercise may modulate autonomic nervous system activity. In particular, AT may lead to a long-term increase in HR variability, increased parasympathetic activity and a reduction in resting sympathetic tone [19].

Future research should include a larger sample size, long-term follow-up, and additional parameters such as lipid profile, orthostatic tolerance, VO₂ max, METs and respiratory quotien t(RQ). The use of dedicated tools to ensure accurate and continuous monitoring; to this end, specific instrumentation (e.g. HR monitor, metabolimeter, etc.) and more specific submaximal incremental tests would further enchance the accuracy and consistency of data collection. For instance, Tawashi et al. showed that the AT prescribing procedure has the ability to improve lipid profile (LDL increase and HDL reduction), cardiopulmonary status (aerobic and respiratory capacity), functional capacity (trunk control and upper-limb motor function), orthostatic tolerance (postural muscle endurance), and on wheelchair mobility performance [20]. The integration of AT into early rehabilitation protocols may offer a cost-effective and accessible strategy to promote functional and cardiovascular improvements in individuals with SCI, warranting further clinical investigation.

## Data Availability

All data generated or analysed during this study are included in this published article.

## References

[1] Hodgkiss DD, Bhangu GS, Lunny C, Jutzeler CR, Chiou SY, Walter M, et al. Exercise and aerobic capacity in individuals with spinal cord injury: a systematic review with meta-analysis and meta-regression. PLoS Med. 2023;20:e1004082 10.1371/journal.pmed.1004082. 38011304 10.1371/journal.pmed.1004082 PMC10712898

[2] Piercy KL, Troiano RP, Ballard RM, Carlson SA, Fulton JE, Galuska DA, et al. The physical activity guidelines for Americans. JAMA. 2018;320:2020–8. 10.1001/jama.2018.14854. 30418471 10.1001/jama.2018.14854 PMC9582631

[3] Martin Ginis KA, van der Scheer JW, Latimer-Cheung AE, Barrow A, Bourne C, Carruthers P, et al. Evidence-based scientific exercise guidelines for adults with spinal cord injury: an update and a new guideline. Spinal Cord. 2018;56:308–21. 10.1038/s41393-017-0017-3. 29070812 10.1038/s41393-017-0017-3

[4] Kim DI, Lee J, Park H, Jeon JY. The relationship between physical activity levels and mental health in individuals with spinal cord injury in South Korea. Int J Env Res Public Health. 2020;17:4423 10.3390/ijerph17124423. 32575553 10.3390/ijerph17124423 PMC7344782

[5] Tweedy SM, Beckman EM, Connick MJ, Geraghty TJ, Theisen D, Perret C, et al. Correspondence Re “Evidence-Based scientific exercise guidelines for adults with spinal cord injury: an update and new Guideline”. Spinal Cord. 2018;56:406–8. 10.1038/s41393-017-0052-0. 29348691 10.1038/s41393-017-0052-0

[6] Scheer J, de Groot S, Tepper M, Faber W, Veeger D, van der Woude LH. Low-intensity wheelchair training in inactive people with long-term spinal cord injury: a randomized controlled trial on fitness, wheelchair skill performance and physical activity levels. J RehabilMed. 2016;48:33–42. 10.2340/16501977-2037. 10.2340/16501977-2037 26660337

[7] De Groot S, van der Scheer JW, van der Windt JA, Nauta J, van der Hijden LJC, Luigjes L, et al. Handrim wheelchair training: effects of intensity and duration on physical capacity. Health. 2013;5:9–16. 10.4236/health.2013.56A2003.

[8] De Groot S, de Bruin M, Noomen SP, van derWoude LHV. Mechanical efficiency and propulsion technique after 7 weeks of low-intensity wheelchair training. Clin Biomech. 2008;23:434–41. 10.1016/j.clinbiomech.2007.11.001. 10.1016/j.clinbiomech.2007.11.001 18077065

[9] Van den Berg R, de Groot S, Swart KM, van derWoude LHV. Physical capacity after 7 weeks of low-intensity wheelchair training. Disabil Rehabil. 2010;32:1717–21. 10.3109/09638281003649961. 20187736 10.3109/09638281003649961

[10] Gaspar R, Padula N, Freitas TB, de Oliveira JPJ, Torriani-Pasin C. Physical exercise for individuals with spinal cord injury: systematic review based on the international classification of functioning, disability, and health. J Sport Rehabil. 2019;28:505–16. 10.1123/jsr.2017-0185. 30300056 10.1123/jsr.2017-0185

[11] Carpio-Rivera E, Moncada-Jiménez J, Salazar-Rojas W, Solera-Herrera A. Acute effects of exercise on blood pressure: a meta-analytic investigation. Arq Bras Cardiol. 2016. 10.5935/abc.20160064. 27168471 10.5935/abc.20160064 PMC4914008

[12] American College of Sports Medicine. Exercise testing and prescription guidelines (2nd Italian ed., based on the 10th US ed.). Chieti, Italy: Calzetti Mariucci. 2021.

[13] Burns SP, Tansey KE. The expedited international standards for neurological classification of spinal cord injury (E-ISNCSCI). Spinal Cord. 2020;58:633–4. 10.1038/s41393-020-0462-2. 32249829 10.1038/s41393-020-0462-2

[14] Gagnier JJ, Kienle G, Altman DG, Moher D, Sox H, Riley D, et al. The CARE guidelines: consensus-based clinical case reporting guideline development. GlobAdv Health Med. 2013;2:38–43. 10.7453/gahmj.2013.008. 10.7453/gahmj.2013.008 PMC3833570 24416692

[15] Permadi AW. The benefits of aerobic training for improving quality of life: a critical review of study. Warmadewa Med J. 2019;4:57–60. 10.22225/wmj.4.2.1016.57-60.

[16] Carter JB, Banister EW, Blaber AP. The effect of age and gender on heart rate variability after endurance training. Med Sci Sports Exerc. 2003;35:1333–40. 10.1249/01.MSS.0000079046.01763.8F. 12900687 10.1249/01.MSS.0000079046.01763.8F

[17] Nicolò A, Massaroni C, Schena E, Sacchetti M. The importance of respiratory rate monitoring: from healthcare to sport and exercise. Sensors. 2020;20:6396 10.3390/s20216396. 33182463 10.3390/s20216396 PMC7665156

[18] Sandrow-Feinberg HR, Houlé JD. Exercise after spinal cord injury as an agent for neuroprotection, regeneration and rehabilitation. Brain Res. 2015;1619:12–21. 10.1016/j.brainres.2015.03.052. 25866284 10.1016/j.brainres.2015.03.052 PMC4540698

[19] Hutchinson MJ, Goosey-Tolfrey VL. Rethinking aerobic exercise intensity prescription in adults with spinal cord injury: time to end the use of “moderate to vigorous” intensity?. Spinal Cord. 2022;60:484–90. 10.1038/s41393-021-00733-2. 34880442 10.1038/s41393-021-00733-2 PMC9209328

[20] Tawashy AE, Eng JJ, Krassioukov AV, Miller WC, Sproule S. Aerobic exercise during early rehabilitation for cervical spinal cord injury. Phys Ther. 2010;90:427–37. 10.2522/ptj.20090023. 20093326 10.2522/ptj.20090023

